# 5-year risk of “adult-onset” chronic diseases during childhood and adolescent transitioning for individuals with cerebral palsy

**DOI:** 10.1016/j.pmedr.2022.101933

**Published:** 2022-07-28

**Authors:** Daniel G. Whitney

**Affiliations:** a Department of Physical Medicine and Rehabilitation, University of Michigan, Ann Arbor, USA; b Institute for Healthcare Policy and Innovation, University of Michigan, Ann Arbor, USA

**Keywords:** Cerebral palsy, Children, Chronic disease, Clinical epidemiology

## Abstract

Epidemiologic evidence documenting risk of chronic diseases as children with cerebral palsy age throughout growth is lacking to inform prevention strategies. The objective was to characterize the 5-year risk of chronic diseases that are typically associated with advanced aging among < 1–13 year olds with cerebral palsy and effects by patient-level factors. This retrospective cohort study used nationwide commercial administrative claims from 01/01/2001–12/31/2018 from children < 1–13 years old with ≥ 5 years of mostly continuous insurance enrollment. The 5-year risk of chronic diseases was examined for the entire cohort with and without cerebral palsy and then by baseline age group (<1–2, 3–5, 6–8, 9–11, 12–13 years old), including cardiorespiratory, metabolic, kidney, and liver diseases, cancer, depression, and osteoarthritis. For cerebral palsy, the association between 5-year chronic disease rate and patient-level factors was assessed using Cox regression. Children with (n = 5,559) vs without (n = 2.3 million) cerebral palsy had a higher 5-year risk of all chronic diseases when comparing the entire cohorts (relative risk, 1.19 to 64.26, all *P* < 0.05) and most chronic diseases when comparing cohorts for each age group. Among children with cerebral palsy, there were effects by gender, co-occurring intellectual disabilities and/or epilepsy, and wheelchair use for some chronic diseases, which can help to identify at-risk children. This study provides novel epidemiologic evidence of 5-year risk of “adult-onset” chronic diseases for children with cerebral palsy during important developmental stages, and associated patient-level factors (to enhance clinical detection). Findings may inform when to implement prevention strategies and who may be more at risk.

## Highlights

• “Adult-onset” chronic diseases may begin in childhood for individuals with CP.
• 5-year disease risk was < 64.3-fold higher for < 1–13 year olds with vs without CP.
• 5-year chronic disease risks were elevated for CP across all developmental stages.
• Patient factors impacted disease risk variably for children with CP.
• Findings may inform when to implement prevention efforts and who is more at-risk.

## 1 Introduction

Cerebral palsy is a pediatric-onset neurological syndrome characterized by an array of motor dysfunction problems (Appleton and Gupta, 2019) leading to low activity and fitness levels, impaired musculoskeletal development, and excess body fat (Whitney et al., 2017, Whitney et al., 2020, Whitney et al., 2019, Henderson et al., 2005, Ryan et al., 2015, Ryan et al., 2015, Moreau et al., 2012, García et al., 2016). Children with cerebral palsy also have heterogeneous and complex healthcare needs, such as management of gastrointestinal complications, sleep problems, seizures, dysphagia, and pain (Novak et al., 2012, Boel et al., 2019, Benfer et al., 2013, Newman et al., 2006, Thomson et al., 2021). Collectively, these factors increase the risk for an accelerated development of an array of chronic diseases that are typically associated with older adults, as well as mental health disorders. For example, epidemiologic studies among 18–30 year olds with cerebral palsy have documented an elevated prevalence of diseases impacting cardiorespiratory, metabolic, kidney, and liver organ systems, as well as osteoarthritis, cancer, and depression (Whitney et al., 2021, Whitney et al., 2018), all of which gets worse with older age (Whitney et al., 2021) and contributes to the high mortality burden for individuals with cerebral palsy (Hemming et al., 2006, Ryan et al., 2019, Stevens et al., 2022, Whitney et al., 2021).

Owing to a variety of risk factors, many chronic diseases can start in childhood for individuals with cerebral palsy. Studies have reported on the prevalence of chronic and co-existing conditions among children with cerebral palsy, advancing knowledge of the extent and breadth of medical complexity for this pediatric population (Berry et al., 2018, Venkateswaran and Shevell, 2008, Murphy et al., 2006, Brooks et al., 2011, Duke et al., 2021, Reyes et al., 2019). However, many of these studies did not examine chronic diseases that are typically associated with aging. This leaves little knowledge of “adult-onset” chronic disease risk during childhood and the adolescent transition, which may be critical periods to implement early medical (e.g., medications, surgery) and behavioral (e.g., lifestyle changes) strategies for chronic disease prevention. Moreover, the greater medical complexity among children with cerebral palsy may enhance vulnerability to organ system dysfunction at different stages of growth, and exacerbate disease risk with increasing age. Therefore, assessing chronic disease risk by distinct stages of development can better inform on when to implement prevention strategies.

Knowledge of which “adult-onset” chronic diseases and mental health disorders emerge during growth could inform early detection and prevention strategies to optimize healthful aging as children with cerebral palsy transition into and throughout their adult years. Therefore, the primary objective of this study was to characterize the 5-year risk of “adult-onset” chronic diseases and depression by narrow age intervals among a large, nationwide sample of children with cerebral palsy and without cerebral palsy for comparison. To enhance clinical interpretations, the secondary objective was to determine if clinically relevant patient-level factors influence the age-related 5-year disease risks among children with cerebral palsy.

## 2 Materials and methods

### 2.1 Design and database

This was a retrospective cohort study that accessed patient-level claims from 01/01/2001–12/31/2018 from Optum’s de-identified Clinformatics® Data Mart Database. This administrative database contains medical claims and has representation across the U.S. (Whitney et al., 2019). To be enrolled in a private payer health plan, the child’s caregiver(s) can be of any age, income, or disability status, but either pays for coverage out-of-pocket or has coverage through their employer. This sample, therefore, may represent a slightly more affluent sector of the population. Findings should be interpreted within the context of this privately insured sample of children.

Claims data are mainly used for billing reimbursement of healthcare services. However, medical conditions can be identified for research purposes by searching for unique codes attached to individual claims. The codes used to identify the variables in this study are presented in Supplementary Table 1. Healthcare services are not “carried forward” in time, meaning that diagnoses, procedures, medications, etc., can only be detected during the sampling period. This is one of the reasons why many research studies using claims data have an extended sampling period to provide sufficient time to identify variables of interest based on healthcare encounters. In addition to the healthcare services, Optum allows researchers access to 1 of 3 additional data tables, but not more than 1 to maintain patient de-identification. This study was part of a larger study that accessed death information, and therefore does not have access to information relating to socio-economic status.

Data are de-identified prior to administration to researchers and so the University Institutional Review Board approved this study as non-regulated as patient consent was not required.

### 2.2 Cohort selection

**Supplementary figure 1:**
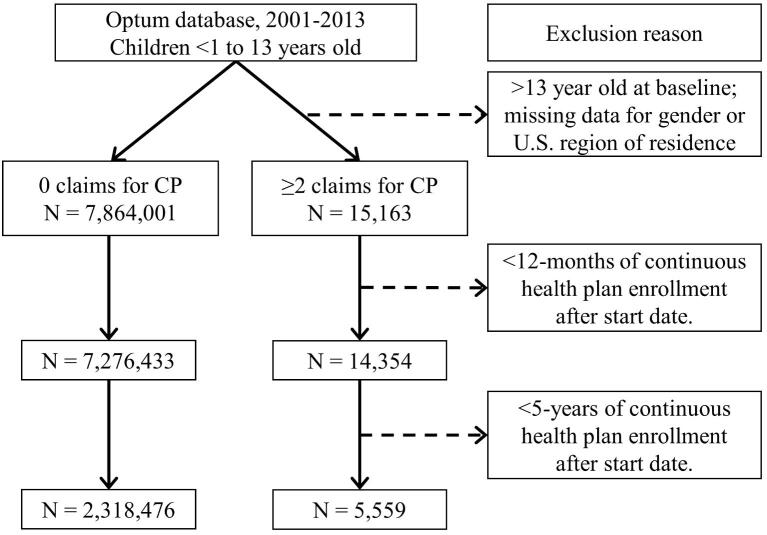


A flow chart of the sample size based on inclusion/exclusion criteria is presented in Supplementary Fig. 1. Children aged < 1 to 13 years of age by 12/31/2013 and with mostly continuous health plan enrollment for ≥ 5 years were eligible for analysis. Requiring full continuous enrollment for ≥ 5 years can lead to selection bias that can be partially avoided. Therefore, to be eligible for analysis, this study allowed for ≤ 3 gaps in health plan enrollment during the 5-year period, where each gap lasted < 3-months and was separated by ≥ 6-months of continuous health plan enrollment. This approach attempted to optimize cohort representation (to mitigate selection bias) and sufficient time to identify diseases (to mitigate detection bias).

The cohort with cerebral palsy was identified using ≥ 2 claims with a relevant code for cerebral palsy, such that each claim for cerebral palsy was on a separate day within 12-months of one another. Using the full 5-year period to identify cerebral palsy mitigates early detection bias, as some children may not get a cerebral palsy diagnosis until 2 or 3 years of age (Hubermann et al., 2016). The cohort without cerebral palsy was defined as children with 0 claims for cerebral palsy over the 5-year period.

### 2.3 Chronic diseases

Chronic diseases were selected based on their relevance to aging with cerebral palsy, ability to detect in claims databases, and diseases that are typically associated with advanced aging and overlooked in pediatric cerebral palsy research. The “adult-onset” chronic diseases include: cerebrovascular disease (e.g., hemorrhage, infarction, arterial occlusion), cardiac conduction disorders and dysrhythmias (e.g., atrioventricular block, atrial fibrillation), heart failure, chronic obstructive pulmonary disease (e.g., chronic bronchitis, asthma), type 2 diabetes, chronic kidney disease (stage I-V or end stage renal disease), other chronic kidney diseases (i.e., chronic renal sclerosis, chronic glomerulonephritis), hypothyroidism, liver disease (e.g., chronic hepatitis, fibrosis, cirrhosis), metastatic cancer, any malignancy except malignant neoplasm of the skin, depression, and osteoarthritis. A single claim with a pertinent code was used to identify the chronic diseases within the 5-year study period.

### 2.4 Patient-level characteristics

Information on age, gender, race, and U.S. region of residence was retrieved from the year of study entry. Age was examined as age groups to examine risks across developmental stages. Claims data does not provide information about pubertal status, which is more complicated in children with cerebral palsy (Kuperminc et al., 2009, Worley et al., 2002). Therefore, the following age groups are not gender specific but reflect general stages of development consistent with the scope of this work: <1–2 years, 3–5 years, 6–8 years, 9–11 years, and 12–13 years. Co-occurring intellectual disabilities and epilepsy are relatively common for children with cerebral palsy and may increase the risk of chronic diseases (Whitney et al., 2021, Reid et al., 2018). Intellectual disabilities and epilepsy were identified in the same manner as cerebral palsy and a variable was constructed to identify mutually exclusive sub-groups of co-occurring intellectual disabilities and/or epilepsy (Whitney et al., 2021). The type of cerebral palsy was described based on available codes.

As claims data does not provide information about the severity of cerebral palsy, a 3-level variable was constructed based on claims for the presence of a wheelchair or wheelchair accessories over the 5-year period to provide a proxy for non-ambulatory status. Children that had a code for “wheelchair dependence” at any point over the 5-year period were designated as non-ambulatory. Children with evidence of medical equipment for a wheelchair/accessories in two separate years were also designated as non-ambulatory; children with evidence of a wheelchair/accessories in one year were designated as having an unknown ambulatory status. This is because surgeries, for example, can lead to a temporary use of a wheelchair for mobility. A single year to capture wheelchair use could misclassify the non-ambulatory status. Children that had 0 claims for wheelchair/accessories over the 5-year period were designated as ambulatory.

### 2.5 Statistical analysis

Basic descriptive characteristics and 5-year risk of each chronic disease were summarized for the cohorts with and without cerebral palsy. The relative risk (RR) with 95 % confidence intervals (CI) was estimated for each chronic disease variable comparing children with vs without cerebral palsy.

The 5-year risk of each chronic disease was estimated for each age group for children with and without cerebral palsy. Given the number of outcome events when stratified by age groups, the following chronic diseases were combined as a single outcome: cancer included metastatic cancer and/or any malignancy; chronic kidney diseases included chronic kidney disease stage I-V, end stage renal disease, chronic renal sclerosis, or chronic glomerulonephritis.

To determine if patient-level factors alter the relationship between age group and 5-year chronic disease rate, Cox regression models were developed and included the main effect of age group, each factor of interest (i.e., gender, co-occurring intellectual disabilities and/or epilepsy, wheelchair use), and their interaction term in separate models. These models adjusted for these patient-level factors, U.S. region of residence, and the study entry year. If the interaction term was not statistically significant, it was removed and the hazard ratio (HR) was estimated to determine the association between these factors and 5-year chronic disease rate. The proportional hazards assumption was tested for each factor in the models based on the weighted Schoenfeld residuals.

### 2.6 Sensitivity analysis

To determine if the chronic disease outcomes in children with cerebral palsy were occurring mostly among those designated as non-ambulatory, children with cerebral palsy without evidence of wheelchair use (proxy for ambulatory status) were compared to two cohorts: (1) children with cerebral palsy designated as non-ambulatory and (2) children without cerebral palsy.

To examine chronic disease risk over a 5-year period, 8,795 children with cerebral palsy were excluded that had 1–4 years of mostly continuous health plan enrollment (Supplementary Fig. 1). To examine for selection bias, baseline characteristics and 1-year chronic disease risks in the first year of follow-up were summarized and compared between children with cerebral palsy that had 1–4 years vs ≥ 5 years (main cohort) of mostly continuous health plan enrollment. In a Cox regression model, the interaction between group (1–4 years vs ≥ 5 years) with age group was tested to determine if the age-related chronic disease rates differed across cohorts.

Analyses were performed using SAS version 9.4 and *P* < 0.05 was considered statistically significant.

## 3 Results

Of the 2,324,035 children eligible for analysis, 5,559 had cerebral palsy with a prevalence of 2.4 per 1,000. The baseline descriptive characteristics of children with and without cerebral palsy are presented in Table 1. The majority had a study entry year in 2001 (cerebral palsy, 26.3 %; without cerebral palsy, 22.8 %). The proportion ranged from 2.1 % to 8.2 % for the remaining 12 years. The majority had no gaps in health plan enrollment for the 5-year period (cerebral palsy, 86.5 %; without cerebral palsy, 86.4 %).

### 3.1 5-year chronic disease risk for the entire cohorts

The most common chronic disease for children with cerebral palsy was chronic obstructive pulmonary disease (36.1 %), followed by cerebrovascular disease (14.4 %) and cardiac conduction disorders and dysrhythmias (9.6 %); for children without cerebral palsy, it was also chronic obstructive pulmonary disease (21.5 %), followed by depression (3.5 %) and cardiac conduction disorders and dysrhythmias (1.5 %) (Table 2). The RR comparing children with vs without cerebral palsy was significantly elevated for all chronic diseases, ranging from 1.2-fold (depression) to 64.3-fold (cerebrovascular disease) higher (Table 2).

**Table 1:** Baseline descriptive characteristics of children with cerebral palsy (CP) and without CP (w/oCP).

	CP (n = 5,559)	w/oCP (n = 2,318,476)
	% (n)	%
Age, mean (SD)	5.9 (4.2)	6.1 (4.3)
<1–2 years	28.5 (1,584)	27.8
3–5 years	20.9 (1,163)	18.5
6–8 years	18.8 (1,046)	19.4
9–11 years	19.7 (1,096)	20.3
12–13 years	12.1 (670)	13.9

Gender		
Female	43.2 (2,403)	48.9
Male	56.8 (3,156)	51.1

Race		
Asian	2.8 (153)	4.5
Black	8.1 (449)	7.2
Hispanic	7.6 (420)	9.6
White	64.0 (3,556)	61.7
Missing	17.7 (981)	17.0

U.S. region of residence		
West	17.9 (994)	21.7
Midwest	28.0 (1,558)	26.1
South	44.9 (2,497)	42.7
Northeast	9.2 (510)	9.6

Co-occurring intellectual disabilities (ID) and epilepsy (EP)		
Without ID and EP	63.0 (3,503)	99.2
ID	5.8 (322)	0.2
EP	21.6 (1,201)	0.6
ID + EP	9.6 (533)	<0.1

Wheelchair use		
No wheelchair	64.3 (3,573)	99.7
Wheelchair use in 2 + years	17.0 (944)	0.1
Wheelchair use in one year only	18.7 (1,042)	0.2

Type of CP		
Spastic		
Quadriplegia	22.5 (1,252)	–
Diplegia	22.3 (1,238)	–
Hemiplegia	20.9 (1,162)	–
Athetoid	0.6 (31)	–
Other/unspecified	33.8 (1,876)	–

SD, standard deviation.

**Table 2:** 5-year risk and relative risk (RR) of chronic diseases among children with cerebral palsy (CP) and without CP (w/oCP).

	CP (n = 5,559)	w/oCP (n = 2,318,476)	
	% (n)	% (n)	RR (95 % CI)
Cerebrovascular disease	14.4 (802)	0.2 (5,205)	64.26 (59.95, 68.89)
Cardiac conduction disorders and dysrhythmias	9.6 (535)	1.5 (34,488)	6.47 (5.97, 7.02)
Heart failure	1.6 (91)	0.1 (2,502)	15.17 (12.33, 18.67)
Chronic obstructive pulmonary disease	36.1 (2,007)	21.5 (497,613)	1.68 (1.62, 1.74)
Type 2 diabetes	1.5 (82)	0.6 (12,975)	2.64 (2.12, 3.27)
Chronic kidney disease stage I-V or end stage renal disease	1.4 (75)	0.1 (2,090)	14.97 (11.91, 18.81)
Other chronic kidney diseases*	0.3 (17)	0.1 (1,092)	6.49 (4.02, 10.48)
Hypothyroidism	3.7 (203)	0.7 (16,868)	5.02 (4.38, 5.75)
Liver disease	2.3 (130)	0.3 (6,802)	7.97 (6.71, 9.46)
Metastatic cancer	0.5 (27)	0.1 (1,148)	9.81 (6.70, 14.35)
Any malignancy except malignant neoplasm of the skin	3.0 (167)	0.5 (10,694)	6.51 (5.60, 7.57)
Depression	4.2 (232)	3.5 (81,274)	1.19 (1.05, 1.35)
Osteoarthritis	0.9 (50)	0.2 (4,173)	5.00 (3.79, 6.60)

CI, confidence interval. *Includes chronic renal sclerosis or chronic glomerulonephritis.

### 3.2 5-year chronic disease risk by age

**Fig. 1:**
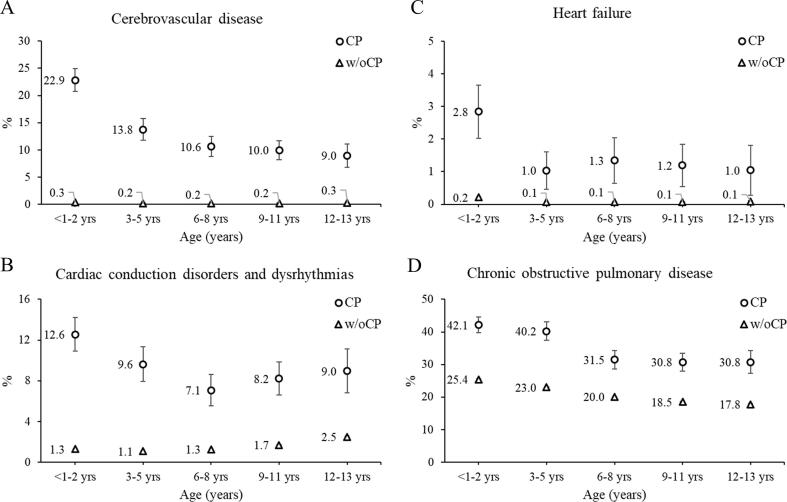
5-year risk of chronic diseases. 5-year risk (%) of (A-D) cardiorespiratory diseases by age group (age determined at baseline) for children with cerebral palsy (CP) and without CP (w/oCP). The marker represents the point estimate (5-year risk) and the vertical lines represent the 95% confidence interval. Due to the large sample size, the 95% confidence intervals for the cohort without CP are difficult to visualize.

**Fig. 2:**
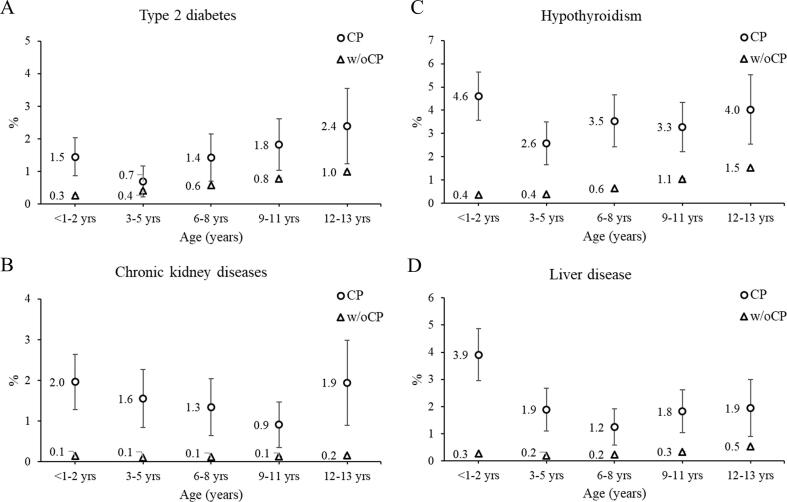
5-year risk of chronic diseases. 5-year risk (%) of (A) type 2 diabetes, (B) chronic kidney diseases (stage I-V, end stage renal disease, chronic renal sclerosis, chronic glomerulonephritis diseases), (C) hypothyroidism, and (D) liver disease by age group (age determined at baseline) for children with cerebral palsy (CP) and without CP (w/oCP). The marker represents the point estimate (5-year risk) and the vertical lines represent the 95% confidence interval. Due to the large sample size, the 95% confidence intervals for the cohort without CP are difficult to visualize.

**Fig. 3:**
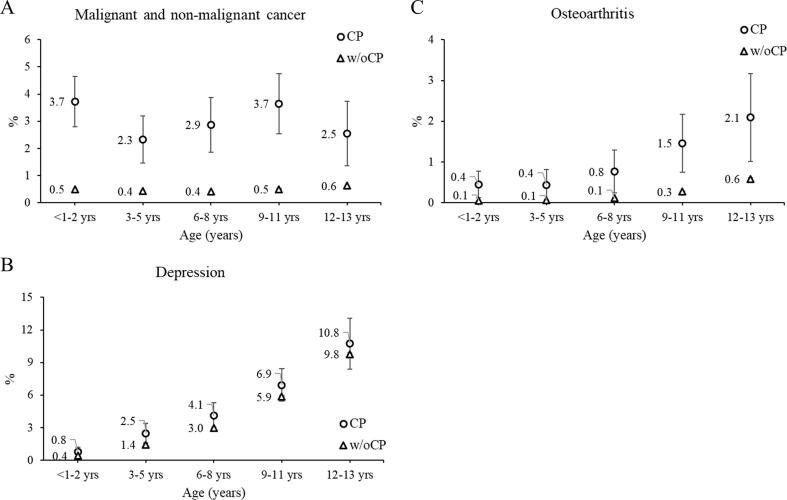
5-year risk of chronic diseases. 5-year risk (%) of (A) cancer, (B) depression, and (C) osteoarthritis by age group (age determined at baseline) for children with cerebral palsy (CP) and without CP (w/oCP). The marker represents the point estimate (5-year risk) and the vertical lines represent the 95% confidence interval. Due to the large sample size, the 95% confidence intervals for the cohort without CP are difficult to visualize.

The 5-year risk of chronic diseases by age group is presented in Fig. 1, Fig. 2, Fig. 3. For both cohorts, there was a high 5-year risk in the youngest age group (<1–2 years) for some of the cardiovascular and other diseases, followed by a lower risk in the next age group (3–5 years). Children with cerebral palsy had higher 5-year risks for each chronic disease across the age groups, except the similar risk of depression across all age groups, type 2 diabetes among 3–5 year olds, and osteoarthritis among < 1–8 year olds.

### 3.3 Patient-level factors associated with 5-year rate of chronic diseases

For the cohort with cerebral palsy, there was no strong evidence of an age group interaction with patient-level factors for any chronic disease outcome. Males vs females with cerebral palsy had a higher HR of chronic obstructive pulmonary disease and a lower HR of liver disease; co-occurring intellectual disabilities and/or epilepsy had a higher HR of all chronic diseases except for depression and osteoarthritis; and evidence of wheelchair use vs non-use had a higher HR of some cardiovascular diseases and chronic obstructive pulmonary disease (Table 3).

**Table 3:** Adjusted association between patient-level factors and 5-year rate of chronic diseases among children with cerebral palsy (CP, n = 5,559).

	Gender	Co-occurring intellectual disabilities (ID) and/or epilepsy (EP)			Wheelchair use	
	Males vs females	ID only vs CP only	EP only vs CP only	ID + EP vs CP only	2 + years vs none	1 year vs none
	HR (95 % CI)	HR (95 % CI)	HR (95 % CI)	HR (95 % CI)	HR (95 % CI)	HR (95 % CI)
Cerebrovascular disease	1.07 (0.93, 1.23)	1.23 (0.87, 1.75)	2.75 (2.35, 3.23)	2.63 (2.10, 3.29)	0.96 (0.79, 1.16)	0.87 (0.72, 1.05)
Cardiac conduction disorders and dysrhythmias	1.02 (0.86, 1.21)	2.41 (1.76, 3.32)	2.00 (1.63, 2.47)	2.77 (2.15, 3.56)	1.59 (1.27, 1.98)	1.41 (1.14, 1.75)
Heart failure	0.92 (0.61, 1.40)	5.44 (2.52, 11.74)	3.84 (2.17, 6.78)	7.52 (4.07, 13.89)**	2.00 (1.19, 3.36)	1.47 (0.86, 2.50)
Chronic obstructive pulmonary disease	1.20 (1.10, 1.31)	1.50 (1.25, 1.79)	1.10 (0.98, 1.23)	1.97 (1.72, 2.25)	1.30 (1.15, 1.47)	1.14 (1.02, 1.28)
Type 2 diabetes	0.99 (0.64, 1.52)	1.90 (0.84, 4.29)	1.60 (0.93, 2.76)	2.64 (1.42, 4.90)	1.00 (0.55, 1.80)	1.04 (0.59, 1.83)
Chronic kidney diseases*	0.69 (0.45, 1.05)	2.15 (0.88, 5.21)	2.55 (1.50, 4.32)	4.79 (2.66, 8.63)	0.95 (0.52, 1.72)	1.26 (0.75, 2.11)
Hypothyroidism	0.96 (0.73, 1.26)	3.35 (2.15, 5.21)	1.70 (1.20, 2.42)	2.80 (1.87, 4.19)	1.06 (0.72, 1.56)	1.35 (0.96, 1.89)
Liver disease	0.65 (0.46, 0.92)	3.07 (1.64, 5.76)	2.62 (1.71, 4.01)	3.37 (2.02, 5.63)	1.86 (1.19, 2.89)	1.39 (0.90, 2.16)
Cancer	1.18 (0.87, 1.60)	2.31 (1.38, 3.87)	1.46 (1.00, 2.12)	2.04 (1.29, 3.23)	1.01 (0.66, 1.55)	1.05 (0.71, 1.55)
Depression	0.83 (0.64, 1.07)	0.30 (0.13, 0.69)	0.74 (0.53, 1.05)	0.48 (0.28, 0.83)	0.78 (0.54, 1.12)	0.93 (0.65, 1.32)
Osteoarthritis	1.05 (0.60, 1.85)	2.03 (0.87, 4.74)	0.71 (0.30, 1.64)	1.48 (0.67, 3.27)	2.01 (1.00, 4.03)	1.73 (0.85, 3.51)

**Supplementary figure 2:**
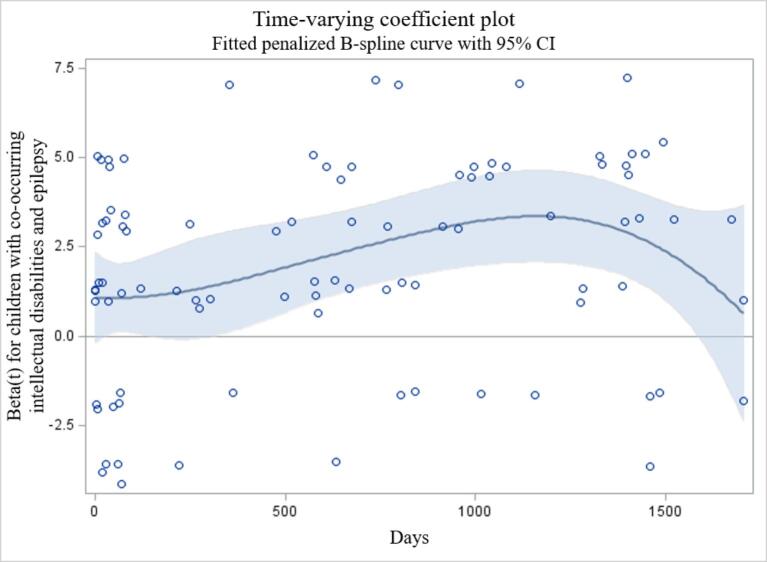


HR, hazard ratio; CI, confidence interval. All models are adjusted for age group, gender, co-occurring intellectual and/or epilepsy, wheelchair use, U.S. region of residence, and the study entry year. *Includes chronic disease stage I-V, end stage renal disease, chronic renal sclerosis, or chronic glomerulonephritis. **Violated the proportional hazards assumption (*P* = 0.039); see Supplementary Fig. 2.

The proportional hazards assumption was violated for children with co-occurring intellectual disabilities and epilepsy when heart failure was the outcome (*P* = 0.039). Upon examining the penalized B-spline curve, which measures the time dependency of the association, the rate of heart failure was elevated throughout the 5-year follow-up, but increased further after ∼ 1-year of follow-up for children with co-occurring intellectual disabilities and epilepsy as compared to children with cerebral palsy only (Supplementary Fig. 2).

### 3.4 Sensitivity analysis

Children with cerebral palsy without evidence of wheelchair use (proxy for ambulatory status) had a higher risk of all chronic diseases compared to children without cerebral palsy (RR range from 1.22 to 61.83, all *P* < 0.05), but similar or lower risk compared to children with cerebral palsy with evidence of wheelchair use (proxy for non-ambulatory status) (RR range from 0.33 to 1.06) (Table 4).

**Table 4:** 5-year risk and relative risk (RR) of chronic diseases among children with cerebral palsy (CP) based on ambulatory (ACP) or non-ambulatory (NACP) status* and children without CP (w/oCP).

	ACP (n = 3,573)	NACP (n = 944)	w/oCP (n = 2,318,476)	ACP vs NACP	ACP vs w/oCP
	% (n)	% (n)	% (n)	RR (95 % CI)	RR (95 % CI)
Cerebrovascular disease	13.9 (496)	15.8 (149)	0.2 (5,205)	0.88 (0.74, 1.04)	61.83 (56.74, 67.39)
Cardiac conduction disorders and dysrhythmias	7.4 (265)	14.4 (136)	1.5 (34,488)	0.51 (0.42, 0.62)	4.99 (4.44, 5.60)
Heart failure	1.0 (37)	3.2 (30)	0.1 (2,502)	0.33 (0.20, 0.52)	9.60 (6.95, 13.25)
Chronic obstructive pulmonary disease	33.3 (1,190)	42.6 (402)	21.5 (497,613)	0.78 (0.72, 0.85)	1.55 (1.48, 1.63)
Type 2 diabetes	1.3 (47)	1.8 (17)	0.6 (12,975)	0.73 (0.42, 1.27)	2.35 (1.77, 3.12)
Chronic kidney diseases**	1.3 (45)	1.8 (17)	0.1 (2,952)	0.70 (0.40, 1.22)	9.89 (7.38, 13.25)
Hypothyroidism	3.1 (109)	4.1 (39)	0.7 (16,868)	0.74 (0.52, 1.06)	4.19 (3.48, 5.05)
Liver disease	1.7 (61)	3.8 (36)	0.3 (6,802)	0.45 (0.30, 0.67)	5.82 (4.53, 7.47)
Cancer	2.9 (105)	3.3 (31)	0.5 (11,042)	0.89 (0.60, 1.33)	6.17 (5.11, 7.46)
Depression	4.3 (153)	4.0 (38)	3.5 (81,274)	1.06 (0.75, 1.51)	1.22 (1.05, 1.43)
Osteoarthritis	0.6 (22)	1.6 (15)	0.2 (4,173)	0.39 (0.20, 0.74)	3.42 (2.25, 5.19)

CI, confidence interval. *Ambulatory and non-ambulatory status determined by no evidence of wheelchair use (ambulatory) or evidence of wheelchair use in ≥ 2 separate years (non-ambulatory). **Includes chronic disease stage I-V, end stage renal disease, chronic renal sclerosis, or chronic glomerulonephritis.

The baseline descriptive characteristics were similar between children with cerebral palsy with 1–4 years (n = 8,795) vs ≥ 5 years (n = 5,559) of mostly continuous insurance enrollment, except for the 36.1 % vs 17.7 % missing data on race, respectively (Supplementary Table 2). The 1-year chronic disease risk was slightly higher for the 1–4 year vs ≥ 5 year cohort for cardiorespiratory diseases (e.g., cerebrovascular disease, 8.1 % vs 6.6 %, respectively; chronic obstructive pulmonary disease, 17.5 % vs 16.3 %, respectively), but relatively similar for all other chronic diseases (Supplementary Table 3). There was no evidence of an interaction between cohort (1–4 vs ≥ 5 years) with age group for the chronic disease outcomes (*P* for interaction ranged from 0.438 to 0.951). This suggests no strong evidence that conclusions from the main analysis are largely subjected to selection bias.

## 4 Discussion

This study documents epidemiologic evidence of the 5-year risk of an array of chronic diseases- that are typically associated with advanced aging- as children with cerebral palsy age throughout childhood and the adolescent transition period. Study findings may inform prevention strategies through increased knowledge of 5-year chronic disease risks by age and the extent of risk elevation/suppression by clinically relevant patient-level factors among children with cerebral palsy.

Interpretation of observations should consider absolute and relative 5-year risks. The relative risk was elevated for entire cohort of children with vs without cerebral palsy for all chronic diseases, ranging from 1.2-fold to 64.3-fold higher. The absolute risks ranged from rare events (0.3 %) to relatively common events (36.1 %), and when examined by age group, 0.1 % to 42.1 %. Some chronic diseases exhibited a high 5-year risk among the youngest age group (<1–2 years), followed by a lower 5-year risk in the next age group(s) for cerebral palsy and non-cerebral palsy cohorts, which is consistent with prior studies in cohorts not isolated to cerebral palsy (Thomson et al., 2021, Tseng, 2010). For example, a nationwide study found that 0–4 year olds had the highest incidence of heart failure that was more than 7-fold higher than older children up to 14 years of age (Tseng, 2010). A study using Medicaid data found that the median age of onset for overlapping outcomes with the current study for children with neurologic impairment was mostly among < 1–2 year olds.

The drop in 5-year risk for 3–5 year olds for some chronic diseases may reflect early and successful treatment. However, interpretation is cautioned as this may reflect clinical investigation for a disease with a resultant billing reimbursement claim for testing procedures, but not necessarily a diagnosis. For cerebrovascular disease, the high risk in < 1–2 year olds may also reflect follow-up care for the initial stroke that may have caused cerebral palsy. In general, claims data can provide evidence, but not confirmation, of conditions. To cast a wide (but not deep) net, this study used a single claim to provide evidence of chronic disease outcomes. For some outcomes, a single claim provides excellent detection for chronic diseases, but other outcomes may benefit from more complex detection algorithms (Birman-Deych et al., 2005, Doktorchik et al., 2019, Nehra et al., 2013). Moreover, some outcomes are subject to poor real-world detection leading to no claims-based evidence, such as the under-recognition of depression. Communication and cognitive impairments are common among children with cerebral palsy, complicating the ability to clinically identify depression. This may explain why the 5-year risk of depression was lower among children with cerebral palsy with vs without co-occurring intellectual disabilities. Nevertheless, the 5-year risk of chronic diseases in this study is similar to or slightly lower than the 6-year risk of chronic diseases among a Medicaid pediatric cohort with neurologic impairment (Thomson et al., 2021). The slightly lower risk for some chronic diseases (e.g., type 2 diabetes) in this privately insured pediatric cohort with cerebral palsy is expected as Medicaid insured children with developmental disabilities have greater medical complexity (Phillips et al., 2019).

There was no strong evidence to suggest that the age-related 5-year chronic disease risk patterns differed by patient factors. The HR can therefore be interpreted as a relatively consistent effect by that patient factor for each age group. For example, the HR for chronic obstructive pulmonary disease was 1.20 for gender, indicating that males had a 20 % higher adjusted disease rate compared to females across all age groups. Not surprisingly, co-occurring intellectual disabilities and/or epilepsy and wheelchair use (to serve as a proxy for ambulatory status) were associated with an increased risk of many chronic diseases, but to varying levels. However, it is important to note that while children with cerebral palsy with evidence of wheelchair use (proxy for non-ambulatory) had the highest risk of chronic diseases, children with cerebral palsy without evidence of wheelchair use (proxy for ambulatory) still had greater 5-year risk of all chronic diseases as compared to children without cerebral palsy. This suggests that early prevention efforts are needed for children with mild to severe forms of cerebral palsy. The effects by patient-level factors presented in Table 3 can be used in combination with the age stratified risks presented in Figure’s 1–3 to identify the age at which children with cerebral palsy may be at a greater risk for specific chronic diseases.

A review of the etiology of and risk factors for chronic diseases among children with cerebral palsy is beyond the scope of this study, and it is important to note that this study did not include other chronic diseases that are relevant to cerebral palsy given the study aim, such as osteopenia or chronic gastroesophageal reflux disease. Further, the criteria for selection of outcomes were based on many factors and is subjective. For example, asthma was part of the criteria to define COPD. While COPD is not well studied in children with cerebral palsy, asthma is present among children and recently shown to be more prevalent among children with vs without cerebral palsy (Xie et al., 2020). The selection of outcomes is not comprehensive, but it does provide novel evidence across many relevant physiologic systems, which is positioned to inform on the broader medical complexity present among children with cerebral palsy.

The generalizability of findings from this privately insured cohort to the broader pediatric population with cerebral palsy is unknown. Based on nationally representative estimates from U.S. children 0–17 years of age, 65.3 % of children with cerebral palsy had private insurance coverage in the year 2016, and there were no differences in parental report of cerebral palsy severity between children with private and public insurance coverage; however, privately insured children with cerebral palsy had poor racial representation (Whitney et al., 2021). In the current study, the prevalence of intellectual disabilities (∼15 %) and epilepsy (∼31 %) is lower than the ∼ 28 % for intellectual disabilities and slightly lower than the ∼ 35–39 % for epilepsy reported from population-based pediatric studies for cerebral palsy (Hollung et al., 2020, Kirby et al., 2011). A population-based study reported ∼ 33 % of children with cerebral palsy as having “limited or no walking” ability (Kirby et al., 2011), which is similar to the ∼ 36 % of children with cerebral palsy in this study with evidence of wheelchair use.

The limitations that directly influence interpretations of this study must be discussed. This study examined a sub-set of medical conditions that children with cerebral palsy can experience; i.e., “adult-onset” chronic diseases. While there are other relevant conditions, this study focused on chronic diseases less studied in children with cerebral palsy to fill clinical knowledge gaps between abnormal health development during childhood and accelerated chronic disease risk during adulthood. Some chronic diseases may have been under-diagnosed leading to conservative estimates, or improved in time thus over-estimating the life-long medical burden. On the other hand, using a single claim may have captured a chronic disease that was being clinically investigated, and eventually not diagnosed. This study constructed a variable to indicate ambulatory status to serve as a proxy for the motor dysfunction severity aspect of cerebral palsy. This approach is not validated and it is unknown how well it captures ambulatory ability. However, the findings align with the hypothesis that non-ambulatory vs ambulatory status had a higher risk of chronic diseases. Lastly, the sensitivity and specificity of identifying cerebral palsy in this privately insured database is not known. A study from Canada reported a high specificity (99.9 %) using a single claim to identify cerebral palsy, but the sensitivity ranged from ∼ 64 % (mild-to-moderate forms of cerebral palsy) to ∼ 86 % (severe forms of cerebral palsy) (Oskoui et al., 2017). A study from the U.S. found that a single year to identify cerebral palsy (≥1 in patient claim or ≥ 2 outpatients claims where each outpatient claim was within 12-months of one another) captured ∼ 80 % of a Medicare adult cohort with cerebral palsy as compared to using 2-years to identify cerebral palsy. The clinical and demographic characteristics of the 20 % of adults with cerebral palsy not captured using a single year overlapped with the 80 % captured in a single year, but were slightly “healthier” as evidenced by a lower multi-morbidity burden (Conner et al., 2022). The current study used an algorithm that increases specificity (≥2 claims within 12-months) and a design that increases sensitivity (using 5-year to identify cerebral palsy). Nevertheless, there may be proportionally less representation by milder forms of cerebral palsy as compared to more severe forms of cerebral palsy in this study. This is anticipated to not largely bias conclusions drawn because, while proportionally less representation, children with milder forms of cerebral palsy are still being represented. The cohort without cerebral palsy was defined as 0 claims for cerebral palsy for the entire 5-year period, enhancing specificity. If there were children with cerebral palsy misclassified into the cohort without cerebral palsy, the findings from this study would reflect conservative estimates. However, given the vast sample size of the cohort without cerebral palsy, the bias introduced by misclassifying children with cerebral palsy as children without cerebral palsy is negligible.

## 5 Conclusion

This study provides novel epidemiologic evidence of 5-year risk of various chronic diseases during growth for children with cerebral palsy, bridging the knowledge gap of abnormal health development during childhood and excess chronic disease risk in the adult years. This study also identified how clinically relevant patient factors associated with chronic disease outcomes by age, thus enhancing clinical detection and informing prevention efforts.

## Funding

This work was supported by the National Institutes of Health (R03HD105589) and the University of Michigan Office of Health Equity and Inclusion Diversity Fund. The funding sources had no role in the design and conduct of the study; collection, management, analysis, and interpretation of the data; preparation, review, or approval of the manuscript; and decision to submit the manuscript for publication.

## CRediT authorship contribution statement

**Daniel G. Whitney:** Conceptualization, Formal analysis, Funding acquisition, Investigation, Methodology, Resources, Writing – original draft, Writing – review & editing.

## Declaration of Competing Interest

The authors declare that they have no known competing financial interests or personal relationships that could have appeared to influence the work reported in this paper.

## Data Availability

The authors do not have permission to share data.
