# Supplementary material for: 5-year risk of “adult-onset” chronic diseases during childhood and adolescent transitioning for individuals with cerebral palsy
Source: Prev Med Rep. 2022 Jul 28;29:101933. doi: 10.1016/j.pmedr.2022.101933 (PMC9344024; doi:10.1016/j.pmedr.2022.101933)
Supplement: Supplementary data 1 [file mmc1.docx]

**Supplementary Table 1.** International Classification of Diseases, Ninth (ICD-9) and Tenth (ICD-10) Revision and Healthcare Common Procedure Coding System (HCPCS) codes to identify variables for this study.

|  | **ICD-9 codes** | **ICD-10 codes** | **HCPCS codes** |
| --- | --- | --- | --- |
| Cerebral palsy | 333.71, 343.0-343.4, 343.8, 343.9 | G80.x |  |
| Epilepsy | 345.x | G40.x |  |
| Intellectual disabilities | 317.x-319.x | F70.x-F73.x, F78.x, F79.x |  |
| Wheelchair and accessories | V46.3 | Z99.3 | E095.x-E099.x, E10.x-E12.x, E22.x, E23.x, E26.x, K000.x-K01.x, K0813-K0899 |
| Cerebrovascular disease | 430.x-437.x | I60.x-I68.x |  |
| Cardiac conduction disorders and dysrhythmias | 426.x, 427.x | I44.x-I49.x |  |
| Heart failure | 402.11, 402.91, 404.11, 404.13, 404.91, 404.93, 428.0-428.9 | I11.0, I13.0, I13.2, I50.x |  |
| Chronic obstructive pulmonary disease | 490.x-496.x | J40.x-J47.x, J67.x |  |
| Type 2 diabetes | 250.0, 250.2, 250.10, 250.12, 250.20, 250.22, 250.30, 250.32, 250.40, 250.42, 250.50, 250.52, 250.62, 250.70, 250.72, 250.80, 250.82, 250.90, 250.92 | E11.x |  |
| Chronic kidney disease stage I-V or end stage renal disease | 403.x, 404.x, 585.x, 586.x | I12.x, I13.x, N18.x, N19.x |  |
| Other chronic kidney diseases, including chronic renal sclerosis or chronic glomerulonephritis | 582.x, 587.x | N03.x, N26.9x |  |
| Hypothyroidism | 243.x-244.2, 244.8, 244.9 | E00.x-E03.x, E89.0 |  |
| Liver disease | 070.22, 070.23, 070.32, 070.33, 070.44, 070.54, 070.6, 070.9, 456.0-456.2, 570.x, 571.x, 572.2-572.8, 573.3, 573.4, 573.8, 573.9, V42.7 | B18.x, K70.0-K70.3, K70.9, K71.3-K71.5, K71.7, K73.x, K74.x, K76.0, K76.2-K76.4, K76.8, K76.9, Z94.4, I85.0, I85.9, I86.4, I98.2, K70.4, K71.1, K72.1, K72.9, K76.5, K76.6, K76.7 |  |
| Metastatic cancer | 196.x-199.1 | C77.x-C80.x |  |
| Any malignancy except malignant neoplasm of the skin | 140.x-172.x, 174.x-195.8, 200.x-208.x, 238.6, V10.00-V10.9 | C00.x-C26.x, C30.x-C34.x, C37.x-C41.x, C43.x, C45.x-C58.x, C60.x-C76.x, C81.x-C85.x, C88.x, C90.x-C97.x |  |
| Depression | 300.4, 301.12, 309.0, 309.1, 311.x | F20.4, F31.3-F31.5, F32.x, F33.x, F34.1, F41.2, F43.2 |  |
| Osteoarthritis | 715.x | M15.x-M19.x |  |

**Supplementary Table 2.** Baseline descriptive characteristics of children with cerebral palsy with 1-4 years vs. ≥5 years of continuous health plan enrollment.

|  | 1-4 years of continuous health plan enrollment (n=8,795) | ≥5 years of continuous health plan enrollment (n=5,559) |
| --- | --- | --- |
|  | % (n) | % (n) |
| **Baseline descriptive characteristics** |  |  |
| Age, mean (SD) | 5.8 (4.1) | 5.9 (4.2) |
| <1-2 years | 27.7 (2,440) | 28.5 (1,584) |
| 3-5 years | 22.7 (1,992) | 20.9 (1,163) |
| 6-8 years | 20.2 (1,777) | 18.8 (1,046) |
| 9-11 years | 18.2 (1,602) | 19.7 (1,096) |
| 12-13 years | 11.2 (984) | 12.1 (670) |
| Gender |  |  |
| Female | 42.5 (3,737) | 43.2 (2,403) |
| Male | 57.5 (5,058) | 56.8 (3,156) |
| Race |  |  |
| Asian | 2.2 (192) | 2.8 (153) |
| Black | 5.9 (521) | 8.1 (449) |
| Hispanic | 6.0 (526) | 7.6 (420) |
| White | 49.8 (4,379) | 64.0 (3,556) |
| Missing | 36.1 (3,177) | 17.7 (981) |
| U.S. region of residence | |  |
| West | 17.0 (1,496) | 17.9 (994) |
| Midwest | 31.8 (2,795) | 28.0 (1,558) |
| South | 42.2 (3,713) | 44.9 (2,497) |
| Northeast | 9.0 (791) | 9.2 (510) |
| Study entry year, median (IQR) | 2005 (2002-2008) | 2004 (2001-2007) |
| Co-occurring intellectual disabilities (ID) and epilepsy (EP) | | |
| Without ID and EP | 68.5 (6,021) | 63.0 (3,503) |
| ID | 4.1 (364) | 5.8 (322) |
| EP | 20.3 (1,788) | 21.6 (1,201) |
| ID+EP | 7.1 (622) | 9.6 (533) |
| Type of CP |  |  |
| Spastic |  |  |
| Quadriplegia | 23.1 (2,029) | 22.5 (1,252) |
| Diplegia | 23.4 (2,060) | 22.3 (1,238) |
| Hemiplegia | 21.5 (1,894) | 20.9 (1,162) |
| Athetoid | 0.4 (34) | 0.6 (31) |
| Other/unspecified | 31.6 (2,778) | 33.8 (1,876) |
| **1-year chronic disease risk** |  |  |
| Cerebrovascular disease | 8.1 (715) | 6.6 (368) |
| Cardiac conduction disorders and dysrhythmias | 4.0 (351) | 3.3 (183) |
| Heart failure | 0.8 (71) | 0.7 (37) |
| Chronic obstructive pulmonary disease | 17.5 (1537) | 16.3 (905) |
| Type 2 diabetes | 0.4 (32) | 0.5 (27) |
| Chronic kidney disease stage I-V or end stage renal disease | 0.7 (58) | 0.6 (35) |
| Other chronic kidney diseases* | <0.3** | <0.3** |
| Hypothyroidism | 1.6 (142) | 1.6 (89) |
| Liver disease | 1.1 (96) | 1.0 (57) |
| Metastatic cancer | <0.3** | <0.3** |
| Any malignancy except malignant neoplasm of the skin | 1.4 (120) | 1.3 (71) |
| Depression | 0.8 (70) | 0.9 (48) |
| Osteoarthritis | 0.2 (13) | 0.2 (13) |

SD, standard deviation; IQR, interquartile range. *Including chronic renal sclerosis or chronic glomerulonephritis. **N<11 and unable to report exact number for patient de-identification purposes.
